# Optimized *Legionella* expression strain for affinity purification of His-tagged membrane proteins eliminates major multimeric contaminant

**DOI:** 10.1128/spectrum.03222-24

**Published:** 2025-05-19

**Authors:** Sukhithasri Vijayrajratnam, Jonasz B. Patkowski, Joshua Khorsandi, Wandy L. Beatty, Shanmugapriya Kannaiah, Ahmet Hasanovic, Tamara J. O'Connor, Tiago R. D. Costa, Joseph P. Vogel

**Affiliations:** 1 Department of Molecular Microbiology, Washington University, St. Louis, Missouri, USA; 2 Centre for Bacterial Resistance Biology, Imperial College London, London, United Kingdom; 3 Department of Biological Chemistry, Johns Hopkins University School of Medicine 1500, Baltimore, Maryland, USA; McGill University, Ste-Anne-de-Bellevue, Quebec, Canada

**Keywords:** polyhistidine epitope tagged proteins, membrane proteins, *Legionella pneumophila*, single particle analysis, cryo-EM, protein purification

## Abstract

Polyhistidine tags are frequently used for isolating proteins through nickel-nitrilotriacetic acid (Ni-NTA) affinity purification. However, proteins rich in histidine can also bind to the Ni-NTA resin, leading to contamination of the purification with undesired proteins. While attempting to purify the *Legionella pneumophila* Dot/Icm type IVB secretion system complex for single particle analysis, we encountered an unknown contaminant protein that bound to the Ni-NTA resin and formed uniform particles visible in negative stain electron microscopy (EM). Mass spectrometry identified this protein, which is encoded by the *Legionella* gene *lpg1596* as a homolog of enoyl-CoA hydratase. Modeling of Lpg1596 revealed surface-exposed histidine clusters, which likely explains its ability to bind to the Ni-NTA resin. Moreover, since enoyl-CoA hydratase homologs are known to multimerize, multimers of Lpg1596 would be large enough to be visible by negative stain EM. To address the problematic issue of Lpg1596 binding to the Ni-NTA resin, we constructed and analyzed a *L. pneumophila* ∆*lpg1596* mutant strain. Notably, Ni-NTA affinity purification of lysates from the ∆*lpg1596* strain did not contain the contaminant protein or generate observable particles. Since the ∆*lpg1596* mutant strain exhibited replication capabilities similar to the wild-type *L. pneumophila* in macrophages, its deletion will likely not affect pathogenesis studies. To facilitate the deletion of *lpg1596* in other *Legionella* strains, we developed a set of natural transformation vectors with various antibiotic resistance markers. In summary, we present a strategy for removing a common Ni-NTA resin binding protein contaminant in *L. pneumophila*, which improves single particle analysis outcomes.

**IMPORTANCE:**

Ni-NTA purifications are a common method for isolating proteins with a His-tag, but they have a drawback: they often enrich unwanted proteins that are rich in histidines, which can contaminate the sample. We identified one such contaminant in *Legionella pneumophila*, a protein with homology to enoyl-CoA hydratases (Lpg1596). This protein binds to the Ni-NTA resin and forms particles that are observable in electron microscope (EM) images, interfering with the analysis. By removing the gene responsible for making this protein (*lpg1596*), the problem was solved, and no unwanted particles appeared in the EM images. The ∆*lpg1596* mutant strain is the first optimized strain for purifying His-tagged membrane proteins from *Legionella*, which can be used for further studies like single particle analysis.

## INTRODUCTION

*Legionella pneumophila* is a bacterial pathogen that causes Legionnaires’ disease, a severe form of pneumonia with a 10% mortality rate (1, 2). Over the last two decades, the incidence of Legionnaires’ disease has been increasing at a steady rate of 9.3% per year in the United States (1). *L. pneumophila* causes disease by surviving and replicating within alveolar macrophages in human lungs (3, 4). The key virulence system for this pathogen is the Dot/Icm Type IVB secretion system (T4BSS), which translocates ~300 effectors into host cells (5–9). The Dot/Icm T4BSS is a large complex consisting of over 30 proteins that form a transmembrane channel extending from the cytoplasm through the inner membrane, periplasmic space, and across the outer membrane (10–15). In the absence of a functional Dot/Icm system, *L. pneumophila* is defective for intracellular growth and, thus, is avirulent (8, 9, 16–18).

Recently, major efforts have been made to determine the structure of T4SSs, including the Dot/Icm T4BSS, and related conjugation systems of plasmids (19–23). For example, a number of labs have obtained *in situ* structures of portions of the Dot/Icm T4BSS system by cryo-electron tomography (cryo-ET) (10–12, 14). These studies have been augmented by single particle analysis (SPA) on two different Dot/Icm T4BSS subcomplexes (24–26). Due to the inability to purify the entire complex using a single epitope tag, a high-resolution structure of the entire Dot/Icm complex has not yet been obtained.

In order to purify the entire Dot/Icm complex, we proposed employing a twin affinity purification approach using two distinct tags on different subcomplexes. Commonly used tags include polyhistidine (27), hemagglutinin (28), c-Myc (29), FLAG (30), Strep (31, 32), or fusions to proteins like glutathione S-transferase (33, 34), maltose-binding protein (35), and calmodulin-binding protein (36, 37). Polyhistidine tags are often favored due to the relative inexpensiveness of the Ni-NTA affinity chromatography and the small size of the motif, which is less likely to interfere with protein function. However, one major confounding problem when purifying polyhistidine-tagged proteins is contamination of the preparations with endogenous proteins that are rich in histidine residues (27, 38). For example, SlyD, GlmS, and Hsp60 are a few *Escherichia coli* histidine-rich proteins known to bind to Ni-NTA resin (39).

We encountered a similar problem while attempting to purify the *Legionella* Dot/Icm T4BSS using an epitope-tagged version of DotO, a membrane-associated ATPase of this specialized secretion system. Passage of *Legionella* lysates over a Ni-NTA resin revealed a prominent ~70 kDa protein that co-purified with DotO. Problematically, the ~70 kDa protein formed particles that could be observed with negative-stained EM, thus obfuscating our single particle analysis. To eliminate this problem, we identified the Ni-NTA binding protein by mass spectrometry and constructed a deletion strain, which can now be used for the purification of polyhistidine-tagged proteins from *Legionella*.

## RESULTS

### Ni-NTA affinity purified samples from solubilized membranes of *L. pneumophila* strains contain homogeneous multimeric particles

**Fig 1:**
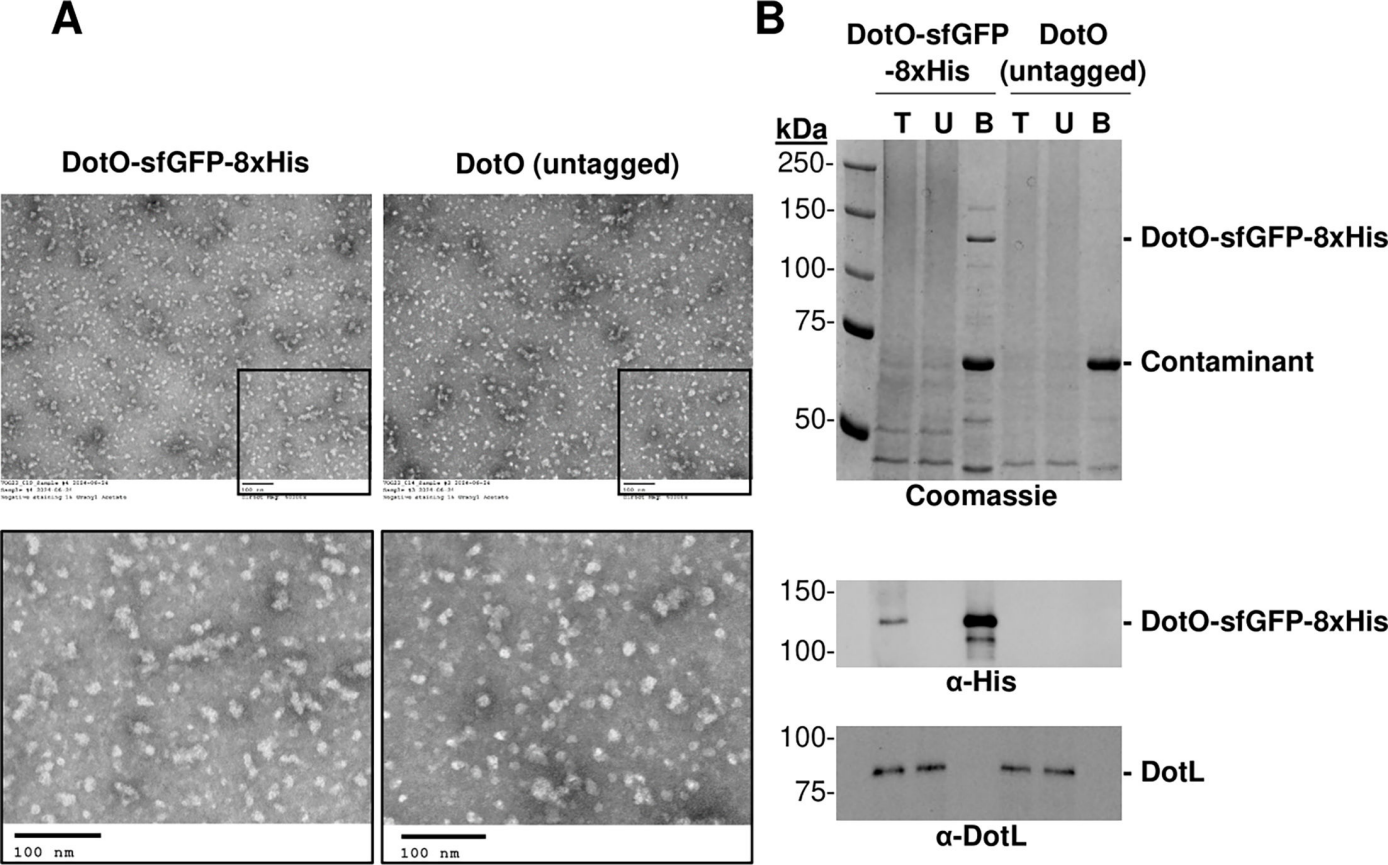
Ni-NTA affinity purifications from *Legionella pneumophila* contain particles, even in the absence of a His-tagged protein. (A) Solubilized membranes from *L. pneumophila* expressing DotO-sfGFP-8xHis or untagged DotO were subjected to Ni-NTA affinity purification. Eluates, after being concentrated using a 100 kDa molecular weight cut-off column, were imaged using negative stained EM (electron microscopy). Upper panels are shown at 60,000× magnification with insets enlarged in the lower panel. Scale bar = 100 nm. (B) Coomassie gel staining and western blot analysis using anti-His and anti-DotL (loading control) antibodies were performed on the following samples: T = total input of solubilized membranes, U = unbound, and B = bound after concentration. Bound samples are 100× overloaded.

As part of our attempt to purify the *Legionella* Dot/Icm complex for SPA, we tagged various Dot/Icm proteins with a polyhistidine tag. We focused our efforts on DotO, as it is a critical, membrane-associated ATPase of the complex (40). Unfortunately, a DotO-8xHis fusion expressed some instability issues, so we turned to a DotO-sfGFP-8xHis fusion, which was more stably expressed. Membranes were harvested from a DotO-sfGFP-8xHis-expressing strain, solubilized, and exposed to Ni-NTA resin, and the bound proteins were eluted using imidazole. The eluate was passed through a 100 kDa molecular weight cut-off concentrator column for a dual purpose: (i) to concentrate the eluate to reduce the volume, and (ii) to remove smaller proteins, thereby size-restricting the sample. However, it is important to note that this step does not eliminate smaller proteins, which form native oligomers or complexes of greater than 100 kDa. The size-restricted, concentrated eluate was then subjected to negative stain-EM analysis to screen for particles and assess their homogeneity (20). Analysis of the preparation revealed abundant, fairly homogeneous particles (Fig. 1A).

The relative homogeneity of the particles was suspicious, as the Dot/Icm complex is very large (over 30 proteins), consists of several subcomplexes (8–12, 15, 24–26, 41), and likely would not appear to be homogeneous if purified using a single tag. As a result, we repeated the purification using solubilized membranes from the isogenic wild-type *L. pneumophila* strain expressing DotO alone and lacking a polyhistidine-tagged protein. Troublingly, the eluate from the control strain showed a similar pattern of particles on negative stain/EM analysis compared to the DotO-sfGFP-8xHis-expressing strain (Fig. 1A). To determine the protein composition of the preparations, samples were separated by SDS-PAGE, and proteins were visualized by staining with Coomassie Blue. A band of the correct size (~130 kDa) was observed from the DotO-sfGFP-8xHis-expressing strain but not from the control strain. This band was recognized by an anti-polyhistidine antibody in a western analysis, and similar to the Coomassie-stained gel, it was not present in the eluate from the control strain, consistent with it being the DotO-sfGFP-8xHis fusion protein. However, we observed a prominent ~70 kDa band in both preparations on the Coomassie-stained gel (Fig. 1B). Since the control strain does not express a polyhistidine-tagged protein, the ~70 kDa band was a native *Legionella* protein, perhaps enriched in histidines, that binds to the Ni-NTA resin, and this “contaminant” was likely responsible for the particles observed via EM analysis.

### ~70 kDa Ni-NTA binding protein is enoyl-CoA hydratase (Lpg1596)

**Fig 2:**
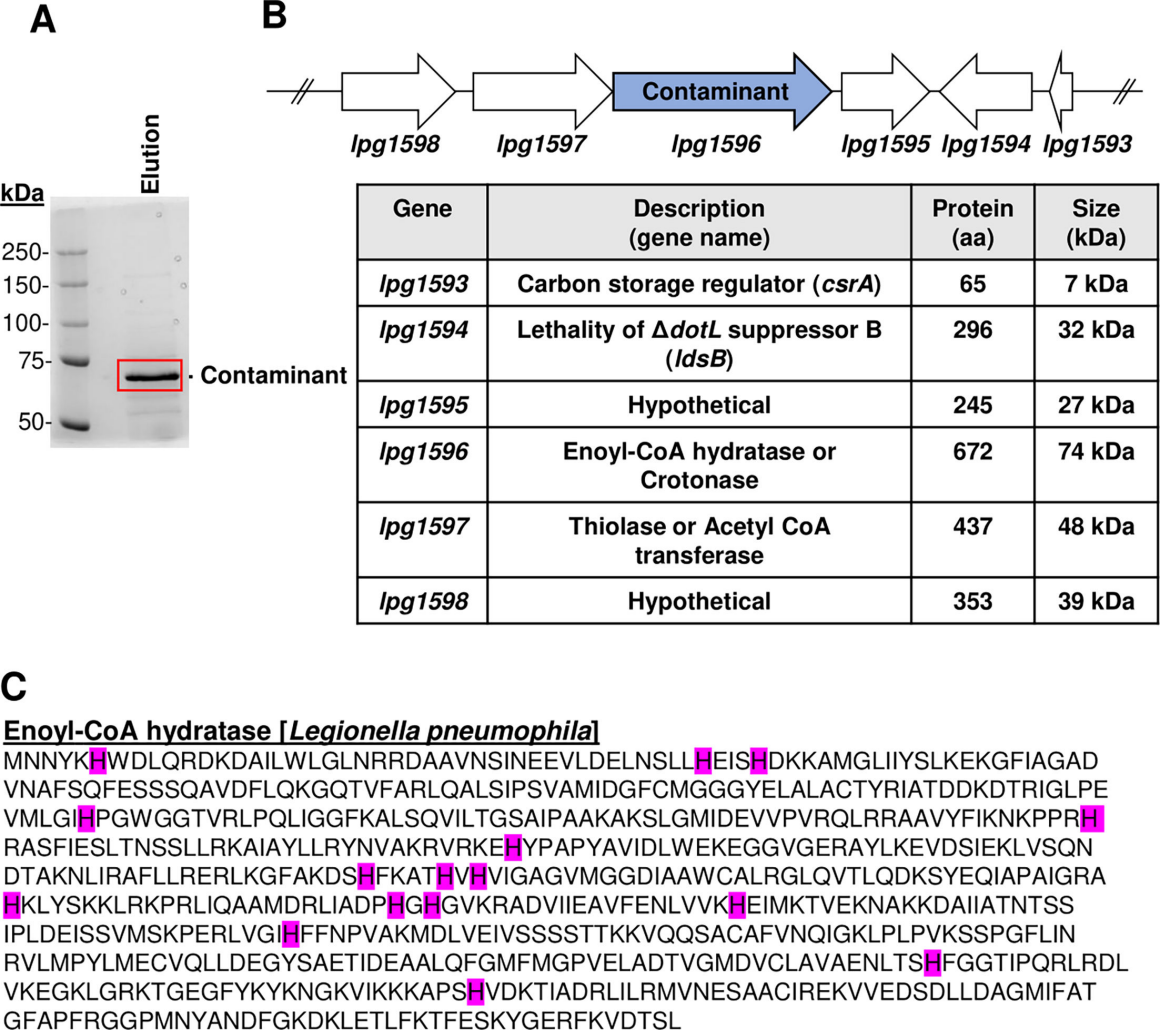
Ni-NTA binding contaminant protein was identified as an enoyl-CoA hydratase homolog encoded by *lpg1596*. (A) Eluate sample was run on an SDS-PAGE gel, and the contaminant band was excised from a Coomassie-stained gel and analyzed by mass spectrometry. (B) *L. pneumophila* (Lp02) chromosomal locus *lpg1598-lpg1593* shown with gene descriptions and protein sizes. (C) Amino acid sequence of Lpg1596 with its 16 histidine residues highlighted in magenta.

To identify the ~70 kDa band, it was excised from the Coomassie-stained gel (Fig. 2A) and subjected to mass spectrometry analysis, which revealed it to be an enoyl-CoA hydratase homolog encoded by the gene *lpg1596* (Fig. 2B). Lpg1596 contains 672 amino acids, which would generate a protein ~70 kDa in size, and *lpg1596* appears to be transcribed in an operon with *lpg1597*, which codes for a related acetyl-CoA transferase (Fig. 2B). Enoyl-CoA hydratases are enzymes of the crotonase family and involved in fatty acid degradation (42–45). All sequenced strains of *L. pneumophila*, which is the most widely studied pathogenic strain of *Legionella* globally, contain highly conserved homologs of Lpg1596. Lpg1596 contains 2.38% histidines (16/672) (Fig. 2C), which is slightly higher than the predicted average of 2% histidines in many proteins (46).

### Sequence analysis and structure-based prediction of histidine residues reveal insights on Lpg1596 binding to Ni-NTA resin

Although we initially assumed that the histidine content in Lpg1596 may favor Ni-NTA binding, a more extensive analysis of the *Legionella* proteome revealed that the histidine content of Lpg1596 was not unusual (Dataset S1, Fig. S1A). Notably, 1,462 of the 2,976 *Legionella* proteins (49.1%) contain a higher percentage of histidines than Lpg1596 ( Dataset S1, Fig. S1A). Moreover, the proteome of the *E. coli* K12 strain contained an average of 2.32% histidines (Dataset S2, Fig. S1B), similar to the 2.4% of histidines observed in the *Legionella* proteome. Therefore, the Ni-NTA binding property of Lpg1596 cannot be explained by histidine enrichment. In addition, Lpg1596 does not contain a long stretch of consecutive histidine residues, a characteristic of many Ni-NTA binding proteins (Fig. 2C).

**Fig 3:**
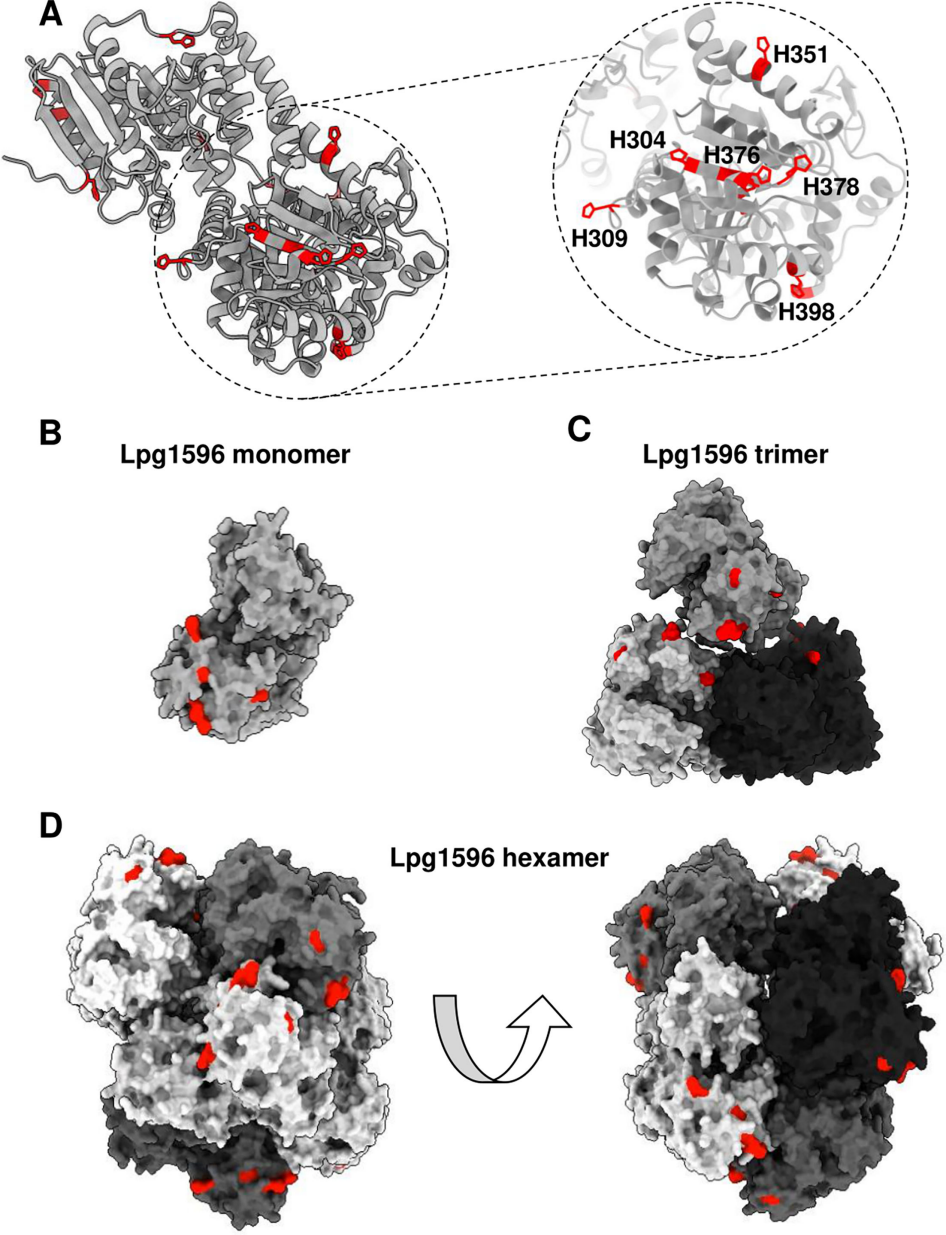
Modeling of Lpg1596 reveals surface-exposed clusters of histidine residues. (A) Lpg1596 (UniProt ID: Q5ZV44) structure was predicted using AlphaFold3. Histidine residues were visualized using ChimeraX and highlighted in red. (B–D) Structure of Lpg1596 shown as a monomer (B), trimer (C), and hexamer (D). The cluster of six histidine residues identified in A is surface-exposed in each form, indicating potential binding sites to the Ni-NTA resin.

Therefore, we hypothesized that the histidines in Lpg1596 may cluster in three dimensions to facilitate binding to the Ni-NTA resin. To test this idea, we modeled the structure of Lpg1596 using AlphaFold (UniProt ID: Q5ZV44) (Fig. 3A) (47). Lpg1596 is predicted to have an N-terminal domain with homology to the enoyl-CoA hydratase/isomerase family and a C-terminal domain with homology to the 3-hydroxyacyl-CoA dehydrogenase family. Modeling revealed that six of the 16 histidine residues in the C-terminus of Lpg1596 (H304, H309, H351, H376, H378, and H398) cluster together. Moreover, these histidine residues are predicted to be surface-exposed and, thus, may serve as an interaction interface with the Ni^2+^ ions of Ni-NTA resin (Fig. 3A and B).

Since crotonase family members, including the *E. coli* YfcX/FadJ (31% sequence identity to Lpg1596), have been reported to exist as trimers or hexamers (dimers of trimers) (42–45), we also modeled Lpg1596 as a trimer and a hexamer (Fig. 3C and D). Notably, the cluster of six histidines remains surface-exposed in either of the potential multimeric forms of Lpg1596 (Fig. 3C and D). Although the cluster of six histidines does not form superclusters in either multimeric form, they do provide additional potential binding sites to the Ni^2+^ ions. As a result, we favor the hypothesis that clustering of surface-exposed histidine residues in Lpg1596 structure mediates binding of the protein to the Ni-NTA resin.

### Removal of *lpg1596* from *Legionella* eliminates the contaminant protein and the multimeric particles

**Fig 4:**
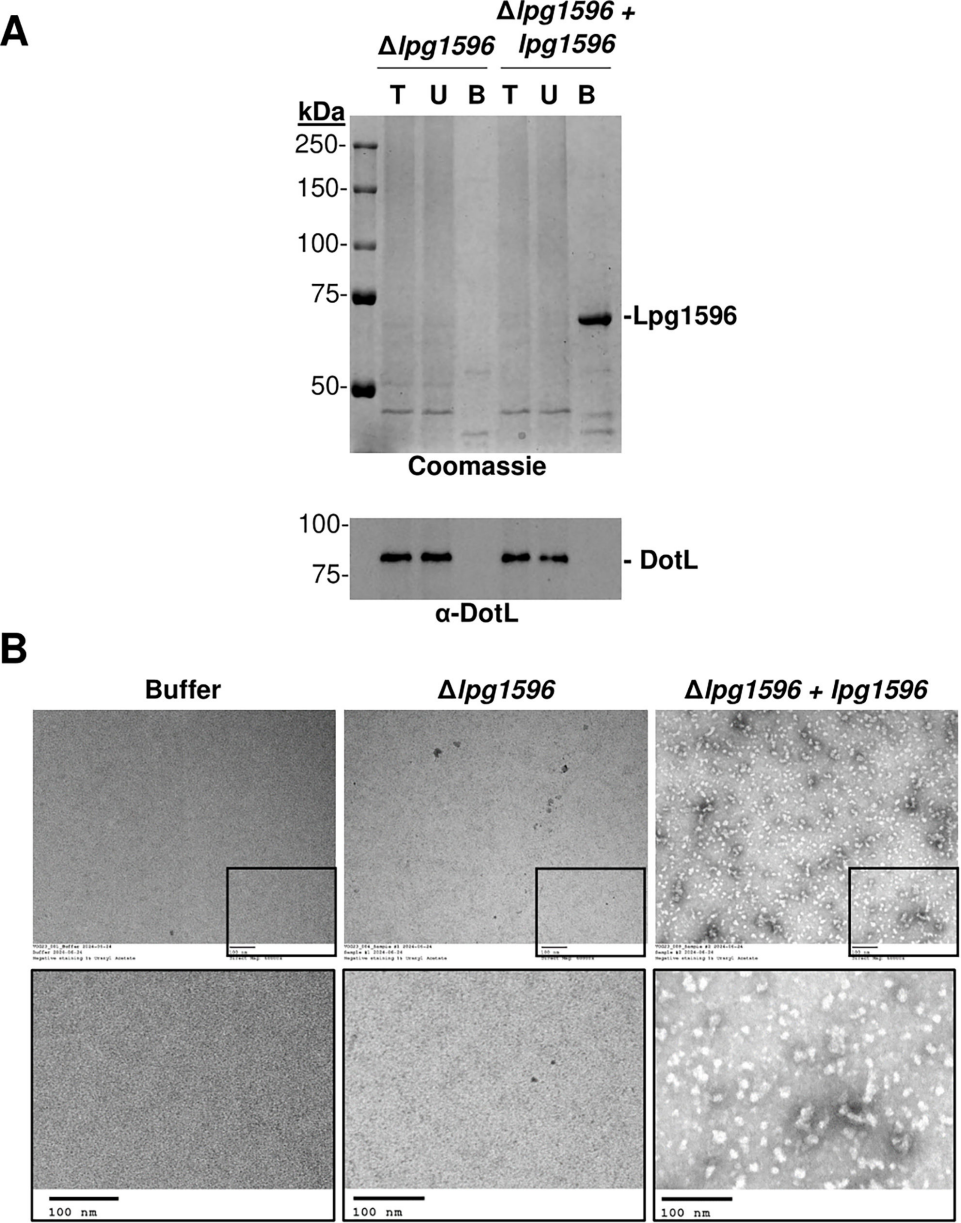
Deletion of *lpg1596* eliminates the suspect contaminant protein and the particles. (A) Solubilized membranes from a ∆*lpg1596* mutant and a ∆*lpg1596* strain expressing Lpg1596 were subjected to Ni-NTA affinity purification. Coomassie gel staining and western blot analysis using anti-DotL antibody (loading control) were performed on the following samples: T = total input of solubilized membranes, U = unbound, and B = bound after concentration. Bound samples are 100× overloaded. (B) Eluate fractions and buffer control were imaged using negative stained EM (electron microscopy). Upper panels are shown at 60,000× magnification with insets enlarged in the lower panel. Scale bar = 100 nm.

Having identified Lpg1596 as the Ni-NTA binding protein, we constructed an in-frame deletion of *lpg1596* (Fig. S2) to determine if the encoded protein was the source of the contaminating particles. Ni-NTA affinity purification of solubilized membranes isolated from the ∆*lpg1596* strain was performed as described above (Fig. 1). In contrast to the eluate from the wild-type control strain (Fig. 1), eluate from the mutant did not contain the prominent ~70 kDa band when analyzed by SDS-PAGE and Coomassie staining (Fig. 4A). In addition, the eluate from Δ*lpg1596* did not reveal any particles on negative staining/EM analysis, similar to buffer control (Fig. 4B). To confirm this result, we examined Ni-NTA purified samples of a Δ*lpg1596* strain complemented with the *lpg1596* gene. The ~70 kDa band re-appeared on the Coomassie gel (Fig. 4A), and particles were observed in negative stained/EM images with the complemented strain (Fig. 4B). In summary, native Lpg1596 is able to bind to the Ni-NTA resin and forms particles, whereas deletion of *lpg1596* eliminates the ~70 kDa contaminant and the particles, thus generating a strain optimized for EM/SPA of histidine-tagged proteins in *L. pneumophila*.

### Single step inactivation of *lpg1596*

Our initial Δ*lpg1596* strain was constructed using two sequential natural transformations, which is a time- and labor-consuming process (Fig. S2) (48). To improve this method, we modified the deletion plasmid to include one of three different antibiotic resistance cassettes that allows for selection of the insertion in one natural transformation reaction. Plasmids pJB8721, pJB8722, and pJB8724 harbor chloramphenicol resistance (CmR), spectinomycin resistance (SpecR), or apramycin resistance (AprR) cassettes, respectively (Fig. S3A and S3B) (49). Using these three plasmids, we were able to inactivate *lpg1596* in one step, and these strains were confirmed to possess the correct antibiotic resistance marker by PCR analysis (Fig. S3C).

### Deletion of *lpg1596* does not affect virulence

**Fig 5:**
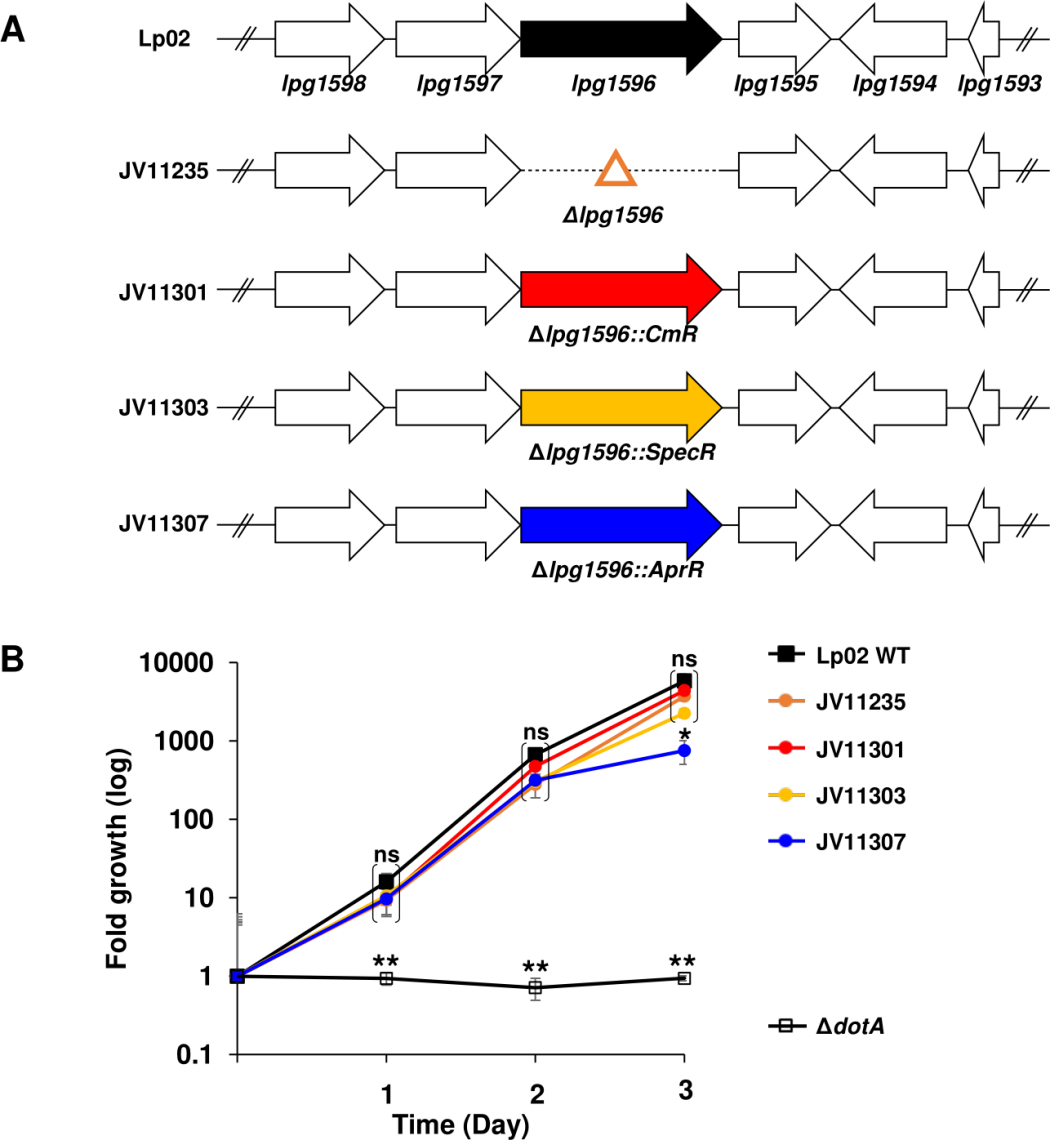
Optimized *Legionella* Δ*lpg1596* strains do not affect intracellular growth in macrophages. (A) Schematic of marked Δ*lpg1596* strains used in this study. (B) Intracellular growth of strains shown in A within U937 cells. Growth was enumerated by counting colony-forming units and graphing fold growth. The wild-type strain (Lp02 WT) and the T4SS-deficient strain (∆*dotA*) were included as positive and negative controls, respectively. Data are the mean ± standard deviation of three biological replicates, each consisting of three technical replicates. ns = not significant, *P < 0.002, **P < 0.005, by two-tailed Student’s *t-*test relative to the Lp02 WT strain.

Since enoyl-CoA hydratases are integral enzymes for the beta-oxidation of fatty acids (50), inactivation of *lpg1596* could affect the ability of *L. pneumophila* to replicate within host cells, thus limiting the utility of this strain. Therefore, to test the effect of Δ*lpg1596* on virulence, we analyzed the ability of the mutant strain to replicate within human monocyte-derived macrophages (U937 cells). As previously published (51, 52), the wild-type strain Lp02 is able to replicate ~5,000-fold over 3 days within U937 cells, whereas a ∆*dotA* mutant, which inactivates the Dot/Icm T4BSS, is unable to grow (Fig. 5). The clean deletion (Δ*lpg1596*) and the chloramphenicol and spectinomycin-marked versions (Δ*lpg1596::CmR* and Δ*lpg1596::SpecR*) showed levels of intracellular growth similar to Lp02, indicating that inactivation of *lpg1596* does not affect growth within a macrophage-like host. The apramycin-marked *lpg1596* deletion strain, Δ*lpg1596::AprR*, exhibited significant growth compared to the ∆*dotA* negative control, although its growth was slightly impaired at day 3 compared to Lp02 (Fig. 5B). However, the original Δ*lpg1596::AprR* mutant (JV11307) and four independently derived insertions all exhibited a corresponding slower growth rate in AYE broth compared to Lp02 and the clean deletion (Δ*lpg1596*) (Fig. S4). Moreover, an Lp02 strain expressing the apramycin resistance cassette (Lp02-AprR) phenocopied the slow growth rate of Δ*lpg1596::AprR* in AYE broth, suggesting that expression of the apramycin cassette is slightly toxic to *Legionella* (Fig. S4B). Consequently, inactivation of *lpg1596* does not affect the ability of *Legionella* to replicate within U937 host cells, indicating that *lpg1596* is not required for optimal virulence within a macrophage-like host.

## DISCUSSION

While attempting to purify the Dot/Icm T4BSS complex for EM/SPA, we discovered that an endogenous *Legionella* protein, Lpg1596, was able to bind to the Ni-NTA resin. This was problematic as the Lpg1596 protein multimerizes, thus forming particles that can be observed on negative stained EM images. Lpg1596 is an enoyl-CoA hydratase, an enzymatic component of the fatty acid degradation pathway (50), although a ∆*lpg1596* strain grows similarly to a wild-type *Legionella* strain in both bacteriological medium and within host cells, indicating it is not an essential gene for virulence. Using lysates from the ∆*lpg1596* mutant, we were able to demonstrate that Ni-NTA purifications no longer contained the ~70 kDa contaminant band we observed on Coomassie gels and lacked particles when viewed by negative stained EM. As a result, the ∆*lpg1596* strain can serve as an optimized strain for the purification of polyhistidine-tagged proteins in *Legionella* for analysis by EM/SPA.

Although Lpg1596 does not have a stretch of consecutive histidine residues, it does contain 16 histidines (2.38% of the protein) (Fig. 2C). Initially, we assumed Lpg1596 may be enriched for histidines, as previous reports indicated that average proteins contain less than 2% histidines (46). However, Lpg1596 turns out to have a median number of histidines when compared to the rest of the *Legionella* proteome (Dataset S1, Fig. S1A). Since the majority of *Legionella* proteins do not bind to the Ni-NTA resin, we hypothesized that a subset of the histidines in Lpg1596 must be surface-exposed and/or cluster in order to be able to mediate binding to the Ni-NTA resin. Modeling of Lpg1596 in a monomeric, trimeric, or hexameric form supported this theory (Fig. 3). Perhaps not too surprising, the *E. coli* proteome contained a similar histidine profile (Dataset S2, Fig. S1B), and, yet, the majority of *E. coli* proteins do not bind to the Ni-NTA resin. Well-known examples of Ni-NTA binding contaminants in *E. coli* include SlyD (10.2% His), GlmS (3.9% His), ArnA/YfbG (4.1% His), and Can/YadF (5.5% His) (39). Like Lpg1596, none of these *E. coli* proteins contain stretches of consecutive histidines, but modeling indicates that all four of these common Ni-NTA binding contaminant proteins also have surface-exposed histidine clusters (Fig. S5).

Previously, researchers have taken a variety of approaches to eliminate these Ni-NTA binding contaminants when purifying polyhistidine-tagged proteins, including deleting the corresponding gene(s) (if it is not an essential gene), mutating surface-exposed histidines, or by tagging the protein with an additional non-polyhistidine tag, thereby allowing its removal on a subsequent resin (52–54). For example, LOBSTR (low background strain) is a modified version of the *E. coli* BL21(DE3) strain created to decrease binding of two of the most common *E. coli* Ni-NTA binding contaminants, ArnA and SlyD (53). Conversely, New England BioLabs (NEB) generated the *E. coli* strain NiCo21(DE3) (NEB #C2529) that expresses a low-affinity variant of GlmS and tagged three other contaminant proteins (SlyD, ArnA, and Can) in a manner that allows their rapid removal by chitin affinity chromatography (54). An alternative approach was to use an *E. coli hfq* mutant to eliminate Hfq in Ni-NTA purifications (55).

However, no such optimized strains exist for most other bacterial species, including *Legionella*. Rather than attempt to eliminate all Ni-NTA binding contaminants, we focused our efforts on developing an improved strain of *L. pneumophila* that could be used for Ni-NTA affinity purification of proteins from bacterial membranes for EM/SPA applications. Since Lpg1596 is the only membrane-associated *Legionella* protein that can bind to the Ni-NTA resin and form particles observable by electron microscopy, use of the Δ*lpg1596* strain solved this specific problem. Moreover, our experimental results demonstrate that Δ*lpg1596* does not affect the intracellular growth of *L. pneumophila* in U937 cells, indicating that *lpg1596* is not critical for virulence. O’Connor and colleagues independently demonstrated that transposon insertions in *lpg1596* do not cause fitness defects in U937 cells or in two commonly used amoebal hosts, *Acanthamoeba castellanii* and *Hartmanella vermiformis* (56). However, they showed that insertions in *lpg1596* may cause minor fitness defects in other amoebal hosts, such as *Acanthamoeba polyphaga* and *Naegleria gruberi* (56). Therefore, the Δ*lpg1596* strain is suitable for use in most *Legionella* experiments.

In summary, while attempting to purify the *Legionella* Dot/Icm T4BSS, we discovered that Lpg1596 is a problematic contaminant for EM/SPA studies when using a polyhistidine-tagged protein, as Lpg1596 binds to the Ni-NTA resin and forms particles observable by negative-stained EM. One option to solving this problem would be to avoid using a polyhistidine tag. However, polyhistidine tags are one of the most commonly used tags due to their small size, which decreases the odds of affecting the protein structure and/or function, and the relatively inexpensive cost of the Ni-NTA affinity resin. Rather than abandoning this tag, we were able to eliminate the troublesome contaminant by deleting the *lpg1596* gene. In order to make this approach more amenable to other researchers, we have also created a set of vectors to efficiently inactivate *lpg1596* in a single step. The resulting optimized strain allows for the contaminant-free Ni-NTA affinity purifications of polyhistidine-tagged membrane proteins expressed in *Legionella*.

## MATERIALS AND METHODS

### Bacterial strains, media, and reagents

*L. pneumophila* strains were grown in yeast extract broth (AYE) or on solid medium consisting of charcoal yeast agar (CYE) both buffered with N-(2-acetamido)-2-aminoethanesulfonic acid. Chloramphenicol (2 µg/mL), spectinomycin (25 µg/mL), apramycin (15 µg/mL), sucrose (5%), and thymidine (T) (100 µg/mL) were added as needed. Strains of *L. pneumophila* used are listed in Table S1. Plasmids and primers used in the study are listed in Table S2 and S3, respectively. Antibodies used in the study are anti-His (THE His monoclonal antibody, Genscript, A00186), polyclonal anti-DotL serum, and secondary antibodies conjugated to horseradish peroxidase [anti-rabbit IgG antibody (Sigma, A0545) and anti-mouse IgG (Sigma, A9917)].

### Large-scale cultures for *L. pneumophila* strains

*L. pneumophila* strains were grown for 2 days on CYET plates, harvested, and resuspended in AYET media, and 7.5E10 bacteria were diluted into 500 mL of media. The culture was incubated on a shaking platform at 37°C for 18–24 h until the OD600 reached early stationary phase (~3.0). The bacteria were harvested by centrifugation, and the bacterial pellets were stored frozen at −20°C.

### Fractionation and harvesting membranes

Frozen pellets containing ~7.5E11 bacteria were thawed and resuspended in 40 mL native lysis buffer (50 mM NaH_2_PO_4_, 300 mM NaCl, 10 mM imidazole pH 7.4) containing 0.5 mg/mL lysozyme and EDTA-free protease inhibitor (Pierce). Samples were incubated on a Nutator for 1 h at 4°C with intermittent vortexing every 15 min. Samples were then passed twice through a Stansted homogenizer at 30,000 psi, followed by several rounds of sonication to ensure complete lysis of the bacteria. The lysate was cleared by two rounds of centrifugation at 23,000 ×*g*. The supernatant (pre-cleared lysate) was subjected to high-speed ultracentrifugation at 100,000 ×*g* for 1 h to harvest the membranes, which were stored at −20°C.

### Solubilization of membranes and Ni-NTA affinity purifications

Purified membrane fraction pellets were thawed and solubilized in 20 mL solubilization buffer [native lysis buffer containing 1% n-Dodecyl-B, D-maltoside (Anatrace), and EDTA-free protease inhibitor (Pierce)] using a Dounce homogenizer to first resuspend the membrane pellet, followed by passage through a 23G needle. The resuspended membranes were then subjected to high-speed ultracentrifugation at 100,000 ×*g* for 1 h to remove any non-solubilized material. The supernatant (solubilized membrane) was incubated with 0.125 mL of His-Pur Ni-NTA resin (ThermoFisher), for 2 h at 4°C. The resin was washed twice using native lysis buffer containing 20 mM imidazole, as a 60 mM imidazole wash failed to remove the Lpg1596 contaminant but started to release the polyhistidine-tagged protein from the resin. The bound proteins were eluted with native lysis buffer containing 250 mM imidazole, concentrated in a Millipore Amicon 100 kDa molecular weight cut-off concentrator, and used for Coomassie gels, western analysis, and electron microscopy (EM).

### Coomassie gel and western analysis

Next, 5 µL of samples was separated using a 7.5% SDS-PAGE gel run for 1 h at 20 mA/gel. For Coomassie gel analysis, gels were stained using Coomassie Blue stain, followed by de-staining with 10% acetic acid. For western analysis, proteins were transferred to polyvinylidene difluoride membrane at 30 V overnight. Membranes were blocked for 1 h in Blotto (5% non-fat dry milk in 1× PBS) and incubated with primary antibody (diluted 1:5,000 in Blotto) for 1 h. Membranes were washed with western wash buffer (1× PBS + 0.05% Tween-20), incubated with secondary antibody diluted 1:100,000 in Blotto for 1 h, washed as above, and then developed with ECL reagent using a Bio Rad ChemiDoc machine.

### Negative staining and transmission electron microscopy

Concentrated eluate samples were fixed with 1% glutaraldehyde (Ted Pella, Inc., Redding CA) and allowed to absorb onto freshly glow discharged formvar/carbon-coated copper grids (Ted Pella, Inc.) for 10 min. Grids were then washed two times in distilled water and stained with 1% aqueous uranyl acetate (Ted Pella, Inc.) for 1 min. Excess liquid was gently wicked off, and grids were allowed to air dry. Samples were viewed on a JEOL 1200 EX transmission electron microscope (JEOL USA, Peabody, MA) equipped with an AMT 8 MP digital camera (Advanced Microscopy Techniques, Woburn, MA). Samples from three independent experiments were analyzed, and representative images are shown in the figures.

### Proteomics and mass spectrometry data analysis

The excised ~70 kDa band from a Coomassie stained gel was washed in 100 mM ammonium bicarbonate (AmBic)/acetonitrile (ACN) and reduced with 10 mM dithiothreitol at 50°C for 30 min. Cysteines were alkylated with 100 mM iodoacetamide in the dark for 30 min at room temperature. The treated gel band was then washed in 100 mM AmBic/ACN prior to digestion with 500 ng trypsin overnight at 37°C. The digested peptides were eluted from the gel at room temperature for 10 min with gentle shaking in 50% ACN/5% FA. The peptide extraction step was repeated using 80% ACN/5% FA and 100% ACN, and all supernatant was saved, then dried in a SpeedVac. After lyophilization, peptides were analyzed by liquid chromatography tandem mass spectrometry using a nanoElute coupled to a timsTOF Pro2 mass spectrometer (Bruker Daltonics). Samples were loaded on an analytical column (75 µm ID × 25 cm C18; IonOpticks) and separated using a linear gradient of solvents A (0.1% formic acid in water) and B (0.1% formic acid in ACN) for over 120 min. Data were searched using Mascot (v.2.8.0 Matrix Science, Boston, MA) against the *L. pneumophila* subsp. *pneumophila* database. The search results were validated with 1% FDR of protein threshold and 90% of peptide threshold using Scaffold v5.3.0 (Proteome Software).

### Strain construction

For generating a clean in-frame deletion of *lpg1596*, a classical two-step natural transformation was performed, as previously described (48). In the first step, *L. pneumophila* wild-type strain Lp02 was naturally transformed with 0.5 µg of the Step#1 plasmid pJB8708 (Δ*lpg1596::sacB/CmR*), and integrants were selected on CYET with 2 µg/mL chloramphenicol. The Step #1 (intermediate) strain JV11570 was then naturally transformed with 0.5 µg of the Step #2 plasmid pJB8706 (Δ*lpg1596*), and integrants were selected on CYET sucrose plates to generate the final Step #2 Δ*lpg1596* strain, JV11235. The single-step marked deletions were generated by natural transformation of the *L. pneumophila* wild-type strain Lp02 using 0.5 µg of plasmids pJB8721 (Δ*lpg1596::CmR*), pJB8722 (Δ*lpg1596::SpecR*), or pJB8724 (Δ*lpg1596::AprR*). Integrants were selected on CYET plates with 2 µg/mL chloramphenicol, 25 µg/mL spectinomycin, or 15 µg/mL apramycin for selection, resulting in the generation of strains JV11301 (Δ*lpg1596::CmR*), JV11303 (Δ*lpg1596::SpecR*), and JV11307 (Δ*lpg1596::AprR*).

### Intracellular growth assays

*L. pneumophila* strains were grown for 2 days on CYET plates, resuspended in sterile water, diluted in RPMI + FBS, and added to pre-differentiated U937 cells at an MOI of 0.1. Infections were allowed to proceed for 1 h, followed by removal of extracellular bacteria by washing. After washing the infected cells, they were lysed in sterile water, and dilutions were plated on CYET plates and incubated for 4 days, then CFUs were counted. Assays were repeated as three independent experiments, each comprising technical triplicates.

### Growth curves in AYE broth media

*L. pneumophila* strains were grown for 2 days on CYET plates and resuspended in AYE broth with supplements to obtain bacterial cultures at a starting OD600 of 0.2. Cultures were grown overnight, and bacterial growth was monitored by measuring the OD600 at the specified time intervals to monitor bacterial growth over 48 h. Assays were repeated as three independent experiments.

### Histidine residue analysis

The frequency of histidine residues was calculated in both the *L. pneumophila* Philadelphia-1 (Lp01) strain, which is the progenitor of the wild-type Lp02 strain (i.e., Lp02 = *thyA* auxotroph of Lp01) (57) and the *E. coli* K12 strain. Calculations of histidine residue frequency were performed across all genome-encoded peptides using Biopython (58), and distribution plots of the percentage of histidines per peptide were generated using the custom Python script available through Github (OConnorLab/LpFreqHIS) and provided at the link: https://github.com/toconnorjhmi/OConnorLab.

### Structural prediction and modeling of proteins

AlphaFold3 (59) was used for structural prediction of Lpg1596 as monomer, trimer, and hexamer. Histidine residues were colored, and structures were visualized using ChimeraX (60). Structures of proteins, SlyD (PDB ID: 2K8I), GlmS (PDB ID: 1JXA), ArnA/YfbG (PDB ID: 1Z7E), and carbonic anhydrase (Can/YadF) (PDB ID: 1I6O) were also visualized using ChimeraX (60).

## SUPPLEMENTAL MATERIAL

Dataset S1 spectrum.03222-24-s0001.xlsx List of proteins in *Legionella pneumophila* Lp01 strain showing percent histidines in their sequence.

Dataset S2 spectrum.03222-24-s0002.xlsx List of proteins in *E. coli* K12 strain showing percent histidines in their sequence.

Supplemental figures and tables spectrum.03222-24-s0003.pdf Fig. S1 to S5 and Tables S1 to S3.

ASM does not own the copyrights to Supplemental Material that may be linked to, or accessed through, an article. The authors have granted ASM a non-exclusive, world-wide license to publish the Supplemental Material files. Please contact the corresponding author directly for reuse.
