# Supplementary material for: Optimized Legionella expression strain for affinity purification of His-tagged membrane proteins eliminates major multimeric contaminant
Source: Microbiol Spectr. 2025 May 19;13(7):e03222-24. doi: 10.1128/spectrum.03222-24 (PMC12211046; doi:10.1128/spectrum.03222-24)
Supplement: Supplemental figures and tables — Fig. S1 to S5 and Tables S1 to S3. [file spectrum.03222-24-s0003.pdf]

Supplementary Information

Optimized *Legionella* expression strain for affinity purification of His-tagged membrane proteins eliminates major multimeric contaminant

Sukhithasri Vijayrajratnam[1], Jonasz B Patkowski[2], Joshua Khorsandi[3], Wandy L Beatty[1], Shanmugapriya Kannaiah[1], Ahmet Hasanovic[1], Tamara J O'Connor[3], Tiago RD Costa[2], Joseph P Vogel[1]

[1]Department of Molecular Microbiology, Washington University, St. Louis, MO 63110.

[2]Centre for Bacterial Resistance Biology, Imperial College London, London, UK.

[3]Department of Biological Chemistry, The Johns Hopkins University School of Medicine, Baltimore, MD, USA

To whom correspondence may be addressed. Email: jvogel@wustl.edu

This PDF file includes:

Figures S1 to S5
Tables S1 to S3
Legends for Datasets S1 and S2
References

Other supporting materials for this manuscript include the following:

Datasets S1 and S2

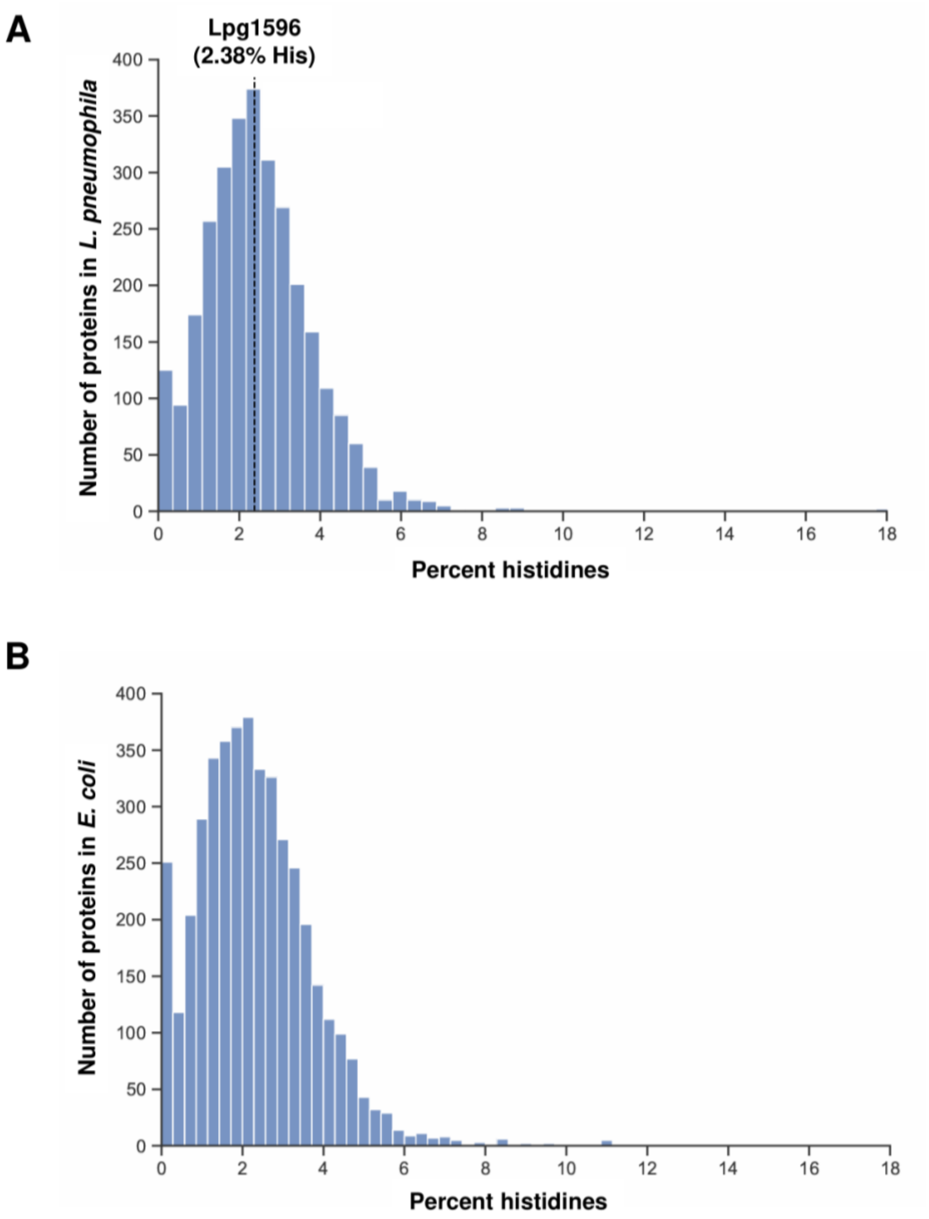

Figure S1. Histidine content of the Legionella proteome. (**A**) Distribution of the 2976 proteins of *Legionella pneumophila* strain Lp01 (progenitor strain of Lp02) based on percent histidines. The position of Lpg1596, with 2.38% histidine residues, is indicated by a dashed line. The percentage of histidines in each *Legionella* protein is provided in Dataset S1. (**B**) Distribution of the 4299 proteins in *E. coli* K12 strain based on percent histidines. The percentage of histidines in each *E. coli* protein is provided in Dataset S2.

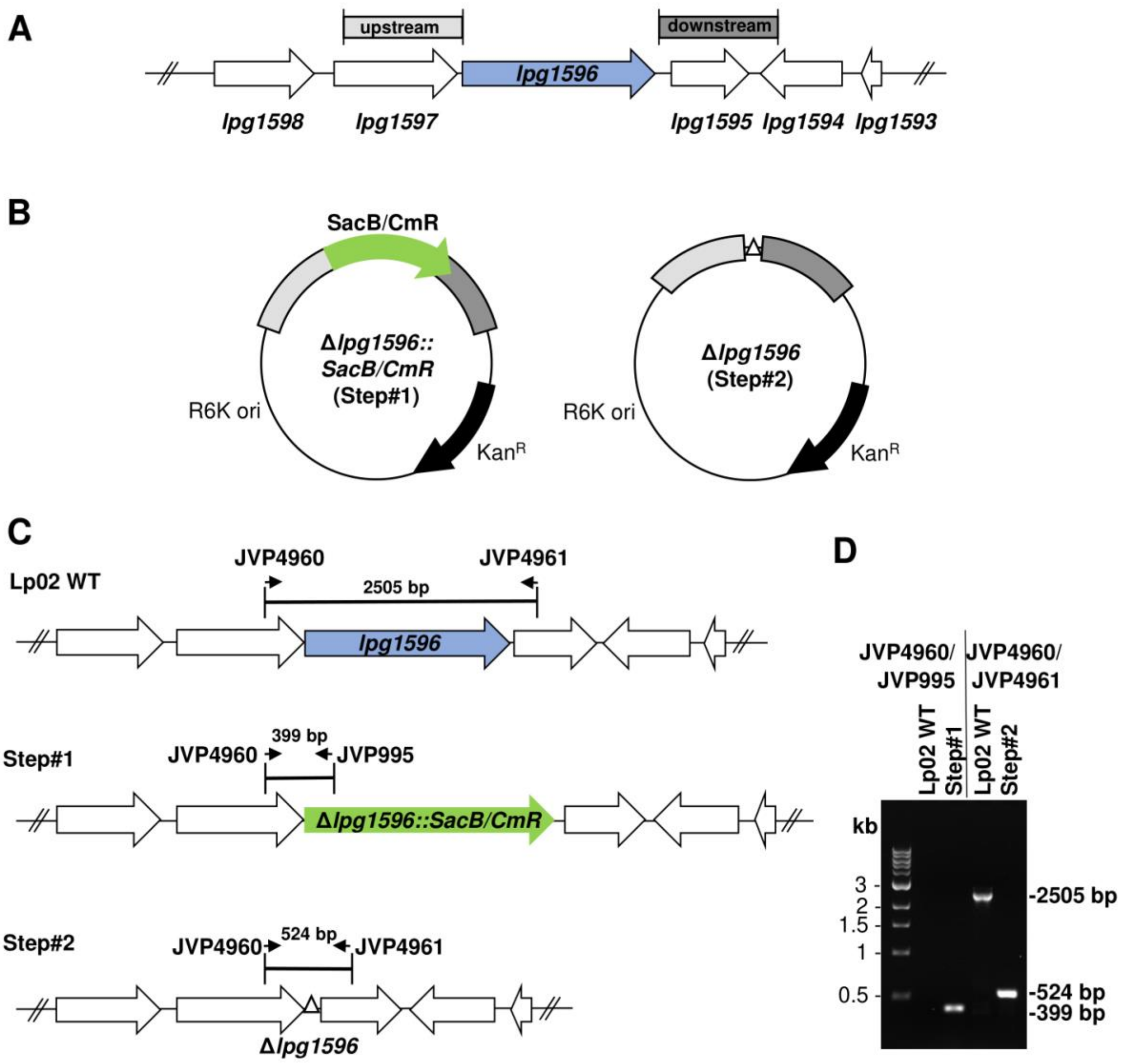

Figure S2. Schematic showing two step construction of Δ*lpg1596* mutant. (**A**) Chromosomal locus of the genes *lpg1598*-*lpg1593* is shown. Regions upstream and downstream of *lpg1596* used in the construction of integration plasmids are denoted as grey bars. (**B**) Step#1 and Step#2 integration plasmids used to construct a deletion of *lpg1596* are shown. (**C**) Construction of Δ*lpg1596* was done by natural transformation in two steps: the *lpg1596* gene was replaced with the *sacB/CmR* cassette (step#1) and then the *sacB/CmR* cassette was replaced by the Δ*lpg1596* (step#2). (**D**) Strains were confirmed by PCR using primer pairs JVP4960/JVP995 and JVP4960/JVP4961. As JVP995 binds to *sacB*, no amplification of DNA was observed with JVP4960/JVP995 and Lp02 WT, as expected.

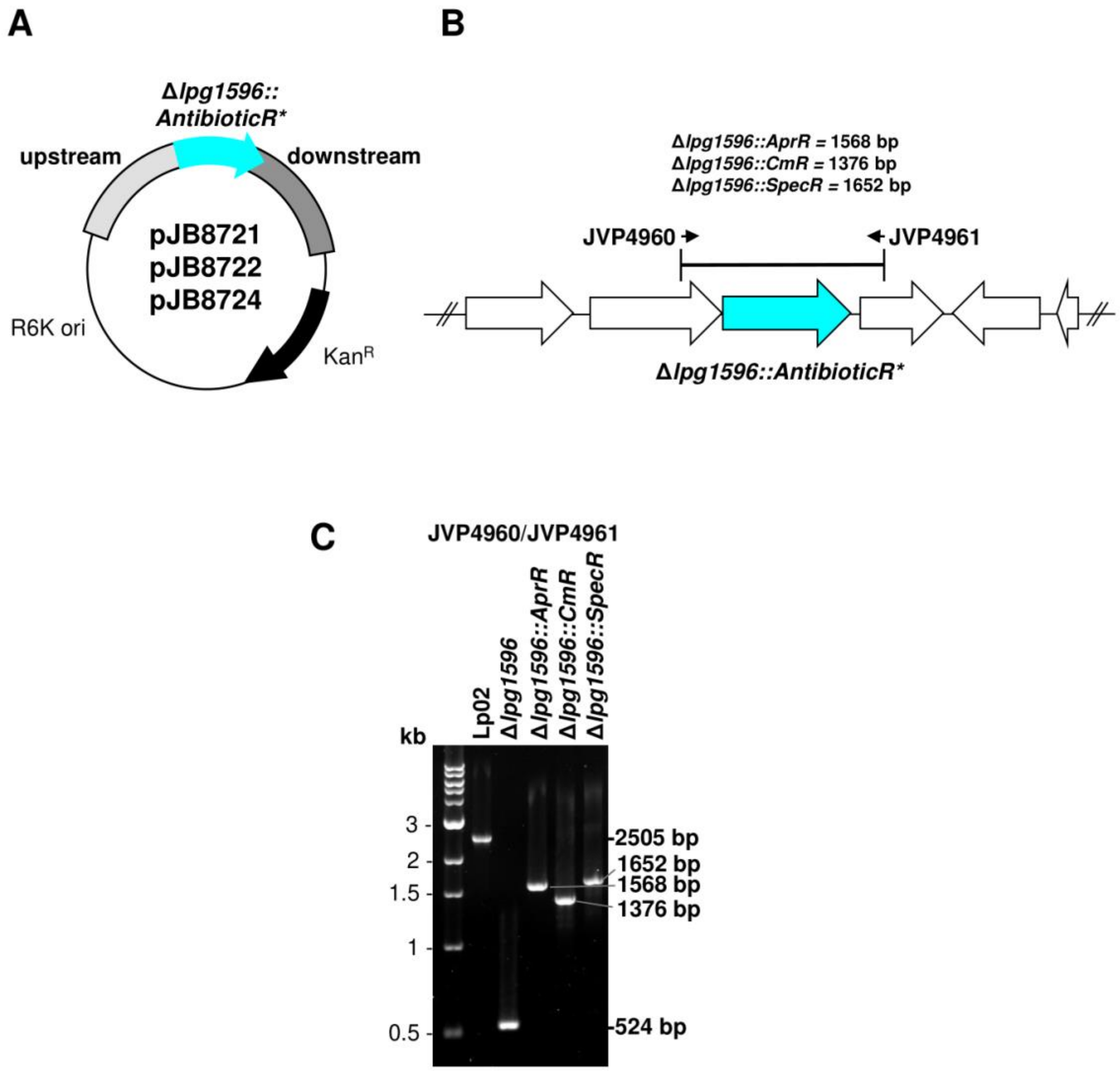

Figure S3. Schematic showing one step construction of antibiotic-marked Δ*lpg1596* mutant. (**A**) Δ*lpg1596 i*ntegration plasmids pJB8721, pJB8722, pJB8724 contain the antibiotic resistance genes for chloramphenicol (*CmR*), spectinomycin (*SpecR*) or apramycin (*AprR*), respectively. (**B**) Schematic of the *lpg1598-lpg1593* chromosomal locus and the Δ*lpg1596::AntibioticR* integrants with the predicted sizes of PCR products shown in **C**. (**C**) Strains were confirmed by PCR using primer pair JVP4960/JVP4961. Lp02 WT and the Δ*lpg1596* mutant show PCR amplicons of 2505 bp and 525 bp, as previously shown in **Fig. S2**.

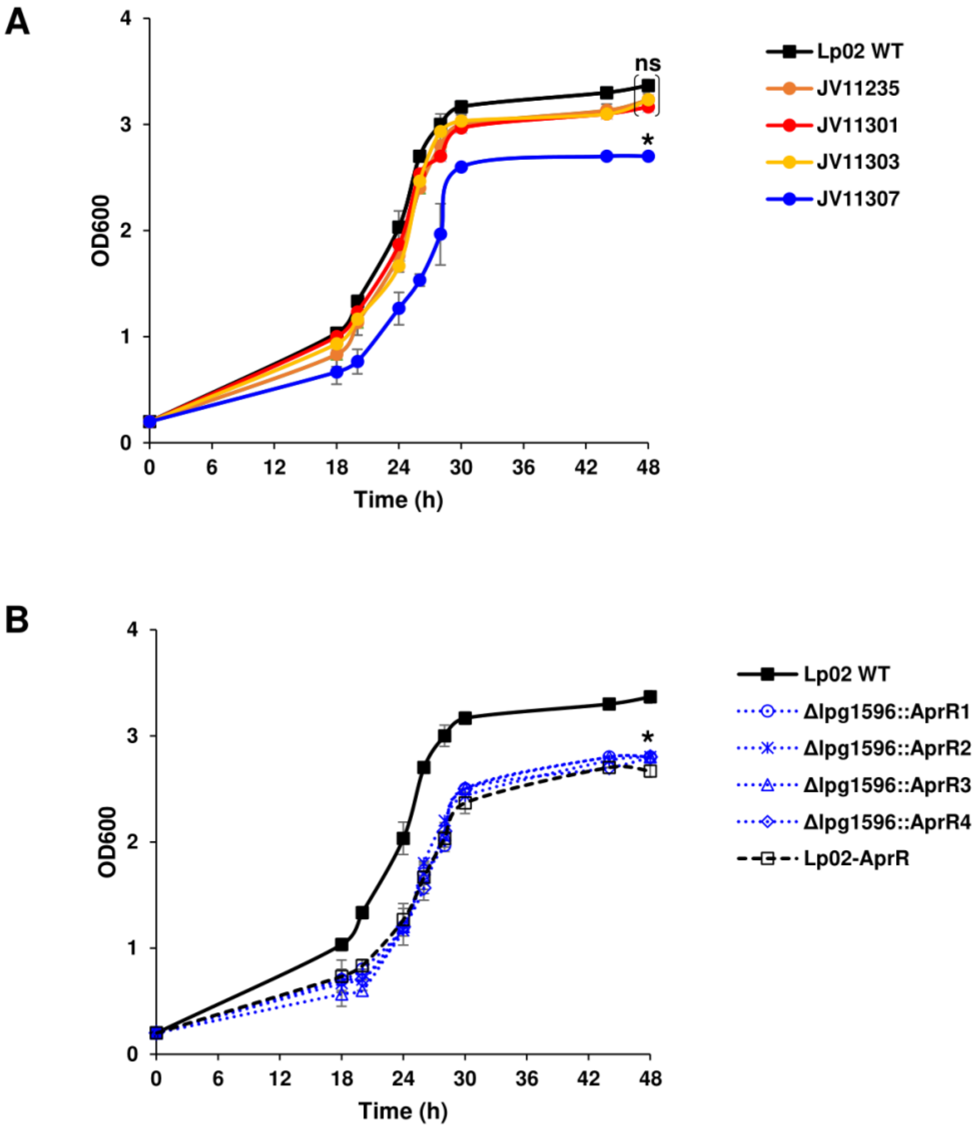

Figure S4. Bacterial growth curve assays of optimized Δ*lpg1596* strains. *L. pneumophila* strains were cultured in in AYE broth media and bacterial growth was monitored by measuring the optical density (OD600) at time intervals through 48 hours. Lp02 was used as positive control. (**A**) Bacterial growth curves for strains used in **Fig. 5B** (intracellular growth assays in U937 cells) are shown. JV11307 (Δ*lpg1596::AprR*) shows a slower growth rate compared to Lp02 and the other optimized Δ*lpg1596* strains. (**B**) Bacterial growth curves for four independently derived isolates of the Δ*lpg1596::AprR* strain, and a control strain of Lp02 expressing the Apramycin resistance cassette (Lp02-AprR) were performed, to confirm the slow growth phenotype. Assays were repeated as three independent experiments. ns = not significant, *P < 0.002 by two-tailed Student t test relative to Lp02 WT.

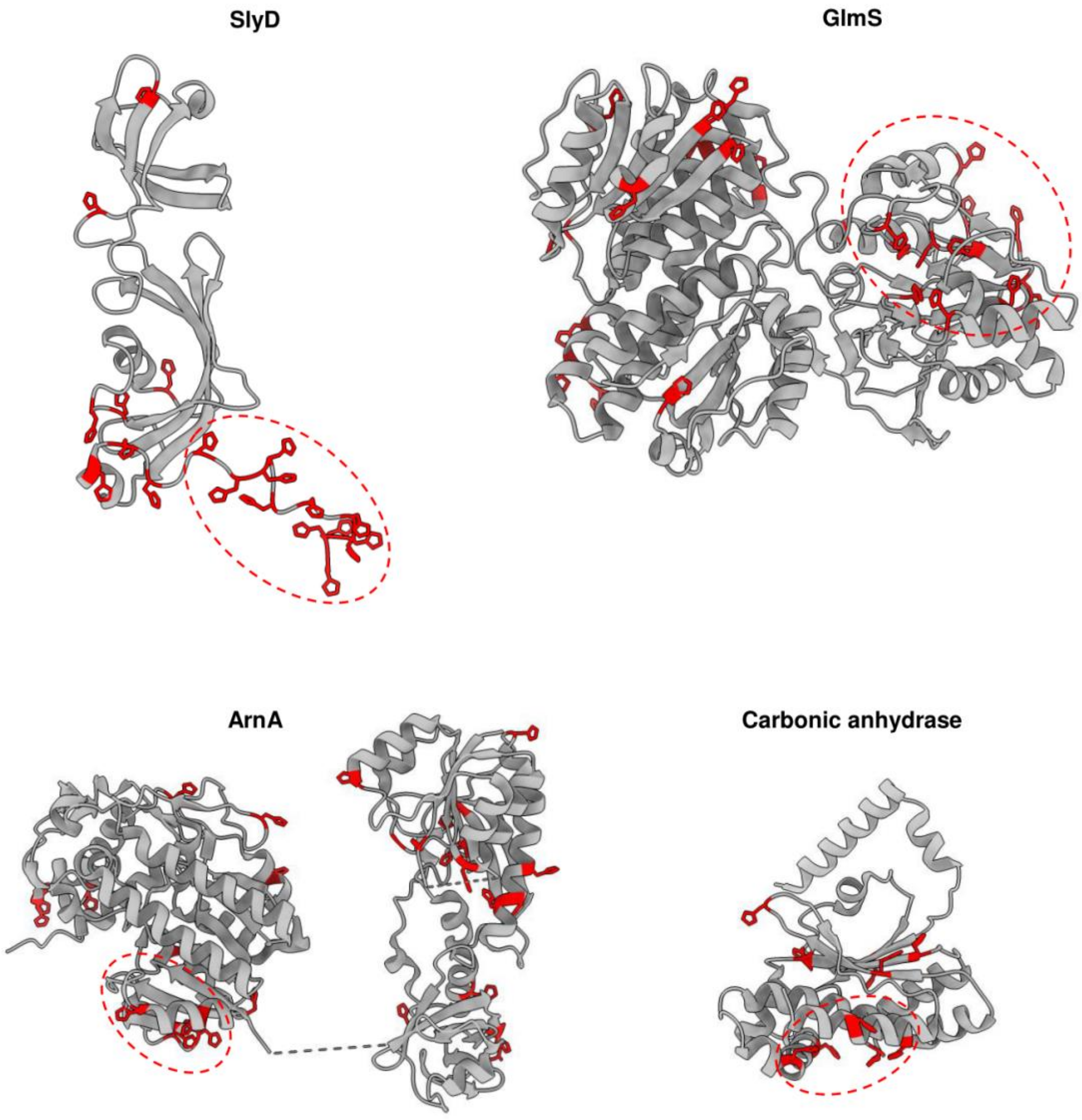

Figure S5. Modeling of four common contaminant proteins in *E. coli* that bind to Ni-NTA resin reveals surface-exposed histidine clusters. Structural models of SlyD (PDB ID: 2K8I), GlmS (PDB ID: 1JXA), ArnA/YfbG (PDB ID: 1Z7E) and carbonic anhydrase (Can/YadF) (PDB ID: 1I6O) were visualized and annotated using ChimeraX. Histidine residues are highlighted in red. Surface-exposed clusters of histidine residues are shown within dashed red ovals.

Table S1: *Legionella pneumophila* strains used in the study

Strain ID	Strain description	Reference
JV10978	DotO-sfGFP-8xHis	This study
JV11235	Δ*lpg1596*	This study
JV11301	Δ*lpg1596::CmR*	This study
JV11303	Δ*lpg1596::SpecR*	This study
JV11307	Δ*lpg1596::AprR*	This study
JV11308	Δ*lpg1596::AprR* 1	This study
JV11591	Δ*lpg1596::AprR* 2	This study
JV11592	Δ*lpg1596::AprR* 3	This study
JV11593	Δ*lpg1596::AprR* 4	This study
JV11570	Δ*lpg1596::sacB/CmR*	This study
JV10435	Lp02-AprR	This study
JV11520	*L. pneumophila* strain Lp02 (wild type)	(1)

Table S2: Plasmids used in the study

Plasmid ID	Plasmid description	Primer pairs/ subcloning strategy	Reference
pJB989	pKRP10 (CmR cassette)	Previously described	(2)
pJB4237	pSR47	Previously described	(3)
pJB6300	pKRP13E (*SpecR* cassette)	Previously described	(2)
pJB7225	X-sfGFP fusion vector	Previously described	(4)
pJB7917	*dotO::sacB/CmR* (Step#1 plasmid)	Previously described	(4)
pJB7934	pBlueScript with *AprR* cassette	JVP3373/JVP3374 to amplify *AprR* from pUV15TetORMApy_mCherry	This study
pJB8540	*dotO-sfGFP-8xHis* (Step#2 plasmid)	JVP3348/JVP3516 to amplify *sfGFP-8xHis* from pJB7225	This study
pJB8706	Δ*lpg1596* (Step#2 plasmid)	JVP3369/JVP3370 and JVP3371/JVP3372 to amplify regions upstream and downstream of *lpg1596*	This study
pJB8708	Δ*lpg1596::sacB/CmR* (Step#1 plasmid)	Subclone *sacB/CmR* fragment from pJB2158 into pJB8706	This study
pJB8721	Δ*lpg1596::CmR*	Subclone *CmR* gene from pJB989 into pJB8706	This study
pJB8722	Δ*lpg1596::SpecR*	Subclone *SpecR* gene from pJB6300 into pJB8706	This study
pJB8724	Δ*lpg1596::AprR*	Subclone *AprR* gene from pJB7934 into pJB8706	This study

Table S3: Primers used in the study

Primer ID	Primer sequence	Restriction sites
JVP3348	GGAGGATCCCGTACCGGTGGCGCGGCACGTAAAGGCGAAGAGCTGTTCAC	BamHI
JVP3369	GACGTCGACCCATTCTGTGGTTCAGATTTGGC	SalI
JVP3370	CCAGGATCCCCAATGTTTATAATTATTCATGTCCTTTCACC	BamHI
JVP3371	CCAGGATCCGTAGATACCAGCTTATAATTTCTCTGC	BamHI
JVP3372	GACGCGGCCGCGAACTACAAAGCAGTTTATTCGCC	NotI
JVP3373	CCATCGACGGATCCACTAGTAAGCTTCTCACGGTAACTGATGCCG	BamHI
JVP3374	CCTTCTAGAGGATCCACTAGTAAGCTTTGTTGCCCCAGCAATCAGC	BamHI
JVP3516	GGATCTAGATCAGTGGTGATGGTGATGGTGATGGTGTTTGTACAGTTCATCCATACCATG	XbaI

Legends for Datasets:

Dataset S1: List of proteins in *Legionella pneumophila* Lp01 strain showing percent histidines in their sequence

Dataset S2: List of proteins in *E. coli* K12 strain showing percent histidines in their sequence

References:

1. Berger KH, Isberg RR. 1993. Two distinct defects in intracellular growth complemented by a single genetic locus in *Legionella pneumophila*. Mol Microbiol 7:7-19.
2. Reece KS, Phillips GJ. 1995. New plasmids carrying antibiotic-resistance cassettes. Gene 165:141-2.
3. Andrews HL, Vogel JP, Isberg RR. 1998. Identification of linked *Legionella pneumophila* genes essential for intracellular growth and evasion of the endocytic pathway. Infect Immun 66:950-8.
4. Vijayrajratnam S, Milek S, Maggi S, Ashen K, Ferrell M, Hasanovic A, Holgerson A, Kannaiah S, Singh M, Ghosal D, Jensen GJ, Vogel JP. 2024. Membrane association and polar localization of the *Legionella pneumophila* T4SS DotO ATPase mediated by two nonredundant receptors. Proc Natl Acad Sci U S A 121:e2401897121.
